# Patient Portal Implementation and Uptake: Qualitative Comparative Case Study

**DOI:** 10.2196/18973

**Published:** 2020-07-27

**Authors:** Melita Avdagovska, Mark Ballermann, Karin Olson, Timothy Graham, Devidas Menon, Tania Stafinski

**Affiliations:** 1 University of Alberta School of Public Health Edmonton, AB Canada; 2 University of Alberta Faculty of Medicine and Dentistry Edmonton, AB Canada; 3 University of Alberta Faculty of Nursing Edmonton, AB Canada; 4 Alberta Health Services Edmonton, AB Canada

**Keywords:** patient portals, information technology, adoption

## Abstract

**Background:**

Giving patients access to their health information is a provincial and national goal, and it is critical to the delivery of patient-centered care. With this shift, patient portals have become more prevalent. In Alberta, the Alberta Health Services piloted a portal (MyChart). There was a need to identify factors that promoted the use of this portal. Furthermore, it was imperative to understand why there was variability in uptake within the various clinics that participated in the pilot.

**Objective:**

This study aims to identify potential factors that could improve the uptake of MyChart from the perspectives of both users and nonusers at pilot sites. We focused on factors that promoted the use of MyChart along with related benefits and barriers to its use, with the intention that this information could be incorporated into the plan for its province-wide implementation.

**Methods:**

A qualitative comparative case study was conducted to determine the feasibility, acceptability, and initial perceptions of users and to identify ways to increase uptake. Semistructured interviews were conducted with 56 participants (27 patients, 21 providers, 4 nonmedical staff, and 4 clinic managers) from 5 clinics. Patients were asked about the impact of MyChart on their health and health care. Providers were asked about the impact on the patient-provider relationship and workflow. Managers were asked about barriers to implementation. The interviews were recorded, transcribed verbatim, and entered into NVivo. A thematic analysis was used to analyze the data.

**Results:**

Results from a comparison of factors related to uptake of MyChart in 5 clinics (2 clinics with high uptake, 1 with moderate uptake, 1 with low uptake, and 1 with no uptake) are reported. Some theoretical constructs in our study, such as intention to use, perceived value, similarity (novelty) of the technology, and patient health needs, were similar to findings published by other research teams. We also identified some new factors associated with uptake, including satisfaction or dissatisfaction with the current status quo, performance expectancy, facilitating conditions, behavioral intentions, and use behavior. All these factors had an impact on the level of uptake in each setting and created different opportunities for end users.

**Conclusions:**

There is limited research on factors that influence the uptake of patient portals. We identified some factors that were consistent with those reported by others but also several new factors that were associated with the update of MyChart, a new patient portal, in the clinics we studied. On the basis of our results, we posit that a shared understanding of the technology among patients, clinicians, and managers, along with dissatisfaction with nonportal-based communications, is foundational and must be addressed for patient portals to support improvements in care.

## Introduction

### Background

The rising costs of health care services and the increased prevalence of chronic conditions necessitate transformative changes in health care delivery. Deliberations on these changes have focused on access to information and the interconnectedness of health information systems [1]. Providing patients secure access to their health information is a provincial [2-5] and national goal in Canada [6,7], and it is considered critical to the delivery of patient- and family-centered care [2-4]. Surprisingly, many portals have underperformed expectations in Canada [8] and in other jurisdictions [9,10]. Although patients today have greater access to their medical information than ever before, there is a need to identify the best way to provide this information and to understand the impact of the provision of medical information to patients on the health care providers.

To address the issues mentioned above, the Alberta Health Services (AHS), a province-wide health delivery organization, piloted a patient portal called eCLINICIAN MyChart (an AHS branding of EpicCare Ambulatory from Epic Systems, 2014 version) between 2016 and 2019 [11]. The goals of the study were to determine whether MyChart would help patients and their families participate actively in the maintenance and monitoring of their health information and to provide information that could be used to scale up the use of MyChart by documenting factors that both hindered and promoted MyChart uptake.

### Objectives

We aimed to accomplish this goal by collecting data from both users and nonusers at the pilot sites. We focused on factors that promoted the use of MyChart along with the related benefits and barriers to its use, with the intention that this information could be incorporated into the plan for its province-wide implementation. To the best of our knowledge, this is the first study that has considered the perspectives not only of patients and health care providers but also of clinic managers and nonmedical staff, such as receptionists, on the impact of patient portal uptake. The timing of data collection began before the introduction of the portal to identify expectations held by health care providers’ practitioners and managers. This approach to data collection was used to identify previously unknown or unanticipated factors that could limit the potential of patient portals for health care systems, including previously obscure power dynamics among clinical leadership. Portals are likely to have systemic groups of nonusers for reasons that have not been previously articulated. In this study, we sought to clarify why portals can, at times, fail to achieve their transformative potential. In parallel with this work, a quantitative survey was conducted on patient views of the portal [12], and the views of the clinicians and managers presented here complement those findings.

## Methods

### Research Question

The research question was as follows: *What factors impacted the differences in uptake of the patient portal MyChart and what elements were deemed crucial for uptake and access for all end users*?

This question was developed by incorporating the principles of *diffusion of innovation* by Rogers [13] and the *theory of technology acceptance* by Davis [14]. Roger’s theory focuses on explaining how and why new ideas and technologies spread, whereas Davis’ s theory focuses on the perceived usefulness of a technology to enhance performance [13,14]. By incorporating these 2 frameworks, we were able to consider the various theoretical approaches relevant when considering the uptake of a patient portal within a publicly funded health care system.

### Ethics Approval

This study received ethics approval from the Research Ethics Board at the University of Alberta (Study ID: MS6_Pro00084135) and operational approval for the clinics included in this study from AHS (OA43157, OA43158, OA43159, and OA43160).

### Design

Using a qualitative comparative case study design [15-17], we performed 56 in-depth semistructured interviews with clinic managers, health care providers, nonmedical providers (receptionists), and patients from 5 clinics that participated in the pilot. Comparative case studies involve the analysis and synthesis of similarities, differences, and patterns across 2 or more cases that share a common focus or goal [15]. In this study, each of the 5 clinics constituted a case. A comparison of cases provided an opportunity to learn more about factors that played a role in the uptake during a real-time patient portal implementation from the standpoint of study participants rather than from existing frameworks.

### Study Setting

Alberta, Canada, has a publicly funded and managed health care system based on the principles of universal access to medically necessary services. AHS [18] is the largest provider of health care services in Alberta, delivering care to more than 4 million people. In 2016, AHS introduced a patient portal called MyChart that was connected to the central electronic medical record system called eCLINICIAN. This was carried out through a pilot where several self-selected clinics decided to participate; however, individual health care providers within the clinics were free to participate or not. MyChart allowed patients to view appointments, medical test results, and medication therapies and to communicate with their health care providers through a computer or an app on a phone or tablet. It was developed by the Epic Systems Corporation (Epic) and customized to fit the needs of AHS. The pilot was led by the eCLINICIAN MyChart Working Group, comprising physicians from both family medicine and various specialties, technology experts, policy makers, and health care administrators.

Following ethics approval, 5 clinics in the pilot were selected for this study based on the number of patients enrolled in MyChart per clinic and the length of time each clinic had been using it. These clinics were considered minimal and safe users of the nonportal functionalities of the Epic product (eg, eCLINICIAN). Three of the clinics were specialist clinics, 1 clinic was a community-based family practice clinic, and 1 clinic was a family practice clinic hosted within an AHS facility.The AHS family practice clinic was selected because although in principle they agreed to participate, they never enrolled any patients. The key features of the cases are outlined in Table 1.

**Table 1:** Case study characteristics.

Details of the clinic	Type of setting	MyChart patient enrollment, n^a^
Clinic 1: medium user of MyChart	Specialty clinic in academic setting	357
Clinic 2: high user of MyChart	Specialty clinic in academic setting	875
Clinic 3: low user of MyChart	Specialty clinic in academic setting	172
Clinic 4: high user of MyChart	General practice clinic in community setting	965
Clinic 5: no users of MyChart	General practice clinic in academic setting	0

^a^As reported in April 2019.

### Sample and Recruitment Strategy

**Figure 1:**
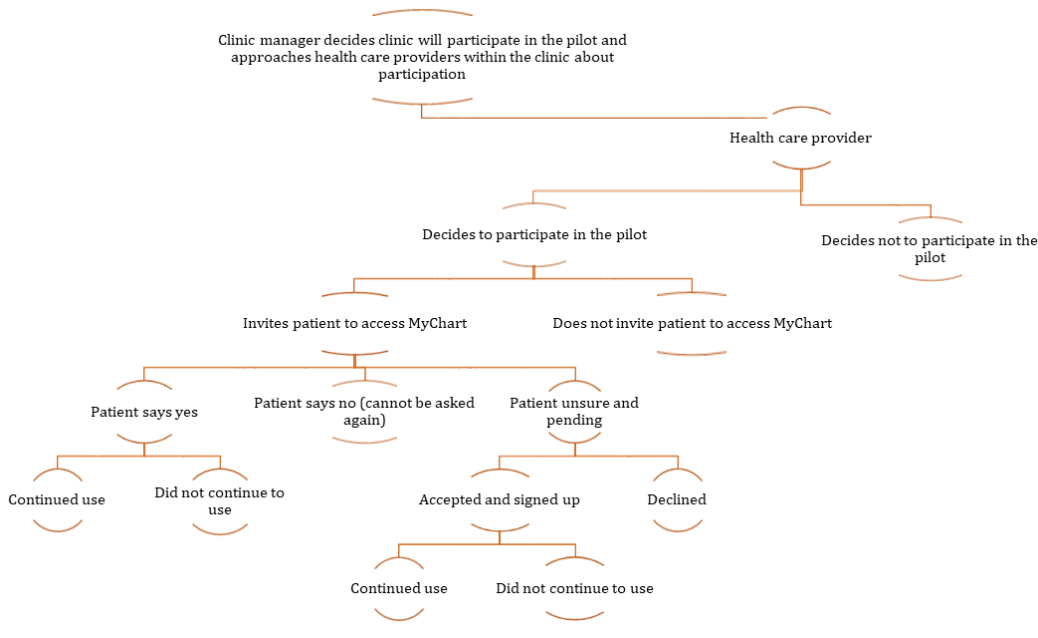
Participant recruitment framework.

Before deciding on the required study sample, the project team had discussions with the MyChart project team and the staff of several clinics in the pilot to understand how its use evolved in each setting. On the basis of those conversations, MA developed a participant recruitment framework (Figure 1), which was reviewed by KO, TS, DM, and MB and then finalized.

On the basis of this framework, we decided to recruit clinic managers and health care providers who did or did not participate in the pilot and patients who used or did not use MyChart, including proxy users. MyChart permits proxy access to family members who provide care to their loved ones.

Clinic managers and health care providers were recruited using a purposive sampling [19] approach. TG contacted each identified clinic, requested an introductory meeting with the clinic manager, and responded to any follow-up concerns. The clinic managers invited health care providers (specialists, general practitioners, and/or nurses) to participate in these introductory meetings. MA developed the presentation and recruitment materials. DM and TS reviewed the materials. MA and MB attended these meetings, described the study, and discussed recruitment strategies for health care providers and patients within each clinic. After answering any questions, MA obtained written consent from health care providers and other clinic staff who were willing to participate in the study. MA set up interviews with clinic managers and health care providers. During the interviews, we identified that only 1 clinic implemented the MyChart web-based booking and canceling appointments function, so all 4 receptionists (nonmedical providers) in that clinic were interviewed.

Patients were recruited using a recruitment card (Multimedia Appendix 1), which was handed out by health care providers at each clinic over a 2-week period. They had provided the card to all patients they saw during those 2 weeks. Patients contacted MA directly if they were interested in participating.

### Data Collection

MA scheduled interviews and explained the study, answered questions, and obtained written consent from those who agreed to participate. The participants then completed a demographic information form and were assigned a deidentifying code used to maintain anonymity and track data. All recruited participants took part in an individual semistructured interview (conducted by MA face-to-face or by telephone) lasting between 30 and 45 min. Notes and reflections were written by MA immediately after each interview. The semistructured interview guides (Multimedia Appendix 2) were developed by MA, based on the research question, and then were reviewed by the other authors and revised based on feedback. To determine whether the interview guides required adjustments, MA evaluated the recordings of the first 2 interviews per group. No changes were made. All interviews were recorded digitally, transcribed verbatim, checked for accuracy, and then uploaded to NVivo 11 (QSR International) to facilitate analysis. Data collection and analysis occurred concurrently until no new dimensions of the participants’ experiences were identified [20].

### Data Analysis

The data collected guided the structure of the subsequent analyses, following the principles of inductive thematic analysis [21-23]. MA cleaned all transcripts and then read each transcript several times, coded transcripts for key ideas related to the research question, and grouped codes into preliminary categories. KO reviewed all codes and categories and discussed them with MA. Preliminary categories across cases and participant groups were organized according to recurring and emerging themes by MA and reviewed by KO and TS. As is common in qualitative research, the findings were built from the data [24]. The findings were then compared with those of other studies.

Several processes were undertaken to maintain the trustworthiness of the data [23], including establishing credibility by recruiting a diverse sample with different and sometimes opposing perspectives and maintaining a comprehensive audit trail in NVivo 11. To ensure transferability of the data, detailed descriptions of the sample and thick descriptions of findings were developed. Analytic rigor was enhanced through regular meetings of the research team to discuss emerging findings. As each interview was completed, all available interviews were reviewed collectively. Data saturation was reached after 25 patient and 20 health care provider interviews, but 2 more patients and 1 more provider were interviewed to ensure that important information was not missed. The demographic data were analyzed descriptively and added as case nodes in NVivo to explore factors associated with the adoption of MyChart.

## Results

### Demographic Characteristics

Our plan was to recruit patients who would fit one of the following categories: user (has access to MyChart and has used it at least once in the past 6 months), declined (offered but declined to sign up), did not continue to use (signed up but did not use), and proxy access user. We were able to recruit only 1 patient who declined and none who did not continue to use, as we had no means of obtaining information about discontinued usage.

We also recruited 1 caregiver who used MyChart under the proxy provisions and several caregivers who used the family member accounts. Table 2 shows the patient respondents’ demographics and reported conditions.

We were able to recruit several health care providers who were nonusers (1 provider elected not to participate in the pilot, 1 medical resident who was not aware that MyChart was part of the clinic where he or she was working, and 3 medical office assistants who were familiar with MyChart; however, as a result of the implementation process for MyChart in that particular clinic, they were not involved directly with MyChart). The specialty physicians were endocrinologists (n=3), neurologists (n=5), and gastroenterologists (n=5). Table 3 shows the health care providers’ and clinic managers’ demographic information.

Overall, 56 participants were recruited (Table 4; to see interview codes and the demographic characteristics per participant group, see Multimedia Appendices 3 and 4).

**Table 2:** Demographics of patient respondents and the reported conditions (n=27).

Characteristics		Value, n (%)
**Gender**		
	Female	17 (62)
	Male	10 (37)
**Age (years)**		
	18-25	1 (3)
	26-35	1 (3)
	36-45	0 (0)
	46-60	16 (59)
	≥61	9 (33)
**Marital status**		
	Never legally married	2 (7)
	Legally married and not separated	18 (66)
	Separated, but still legally married	0 (0)
	Divorced	2 (7)
	Common law	4 (14)
	Widowed	1 (3)
**Education level**		
	Less than high school degree	0 (0)
	High school degree or equivalent	5 (18)
	Some postsecondary education but no degree	7 (25)
	Registered apprenticeship or other trades certificate or diploma	5 (18)
	Associate degree	1 (3)
	Bachelor’s degree	7 (25)
	Graduate degree	0 (0)
	Postgraduate degree	2 (7)
**Employment status**		
	Employed, working ≥40 hours per week	9 (33)
	Employed, working 1-39 hours per week	7 (25)
	Not employed, looking for work	0 (0)
	Not employed, not looking for work	2 (7)
	Retired	4 (14)
	Unable to work	3 (11)
	Self-employed	2 (7)
**Family income (Can $)**		
	<20,000 (US $14,723)	0 (0)
	20,000-34,999 (US $14,723-25,765)	1 (3)
	35,000-49,999 (US $25,766-34,806)	2 (7)
	50,000-74,999 (US $36,807-55,210)	1 (3)
	75,000-99,999 (US $55,211-73,614)	4 (14)
	100,000-149,999 (US $73,615-110,422)	8 (29)
	≥150,000 (US $110,423)	11 (40)
**Chronic condition**		
	Yes^a^	23 (85)
	No	4 (14)
**MyChart users**		
	Yes	25 (92)
	No	2 (7)
	And/or proxy	5 (18)

^a^Chronic conditions reported by the participants included arthritis, Barrett syndrome, bleeding disorder, breast cancer, celiac disease, chronic obstructive pulmonary disease, Crohn disease, depression or anxiety, diabetes type 1, diabetes type 2, high blood pressure, hypothyroid, inflammatory bowel syndrome, irregular heartbeat, Langerhans cell histiocytosis, multiple sclerosis, osteoporosis, prediabetes, psoriasis, relapsing-remitting multiple sclerosis, sleep apnea, systemic mastocytosis, thyroid disease, and ulcerative colitis.

**Table 3:** Demographic information of the health care providers (n=21) and clinic managers (n=4).

Characteristics		Health care providers’ demographics, n (%)	Clinic managers’ demographics, n (%)
**Gender**			
	Female	16 (76)	3 (75)
	Male	5 (23)	1 (25)
**Age (years)**			
	<18	0 (0)	0 (0)
	18-29	0 (0)	0 (0)
	30-39	2 (9)	0 (0)
	40-49	8 (38)	2 (50)
	50-59	8 (38)	1 (25)
	60-64	2 (9)	1 (25)
	≥65	1 (4)	0 (0)
**Type of**			
	Family physician	2 (9)	1 (25)
	Specialty physician	5 (23)	2 (50)
	Registered nurse	8 (38)	1 (25)
	Nurse practitioner	1 (4)	0 (0)
	Medical office assistant	4 (19)	0 (0)
	Resident	1 (4)	0 (0)
**Working at**			
	Academic-based care	14 (66)	3 (75)
	Community-based care	7 (33)	0 (0)
	Both	0 (0)	0 (0)
**In which type of setting do you work**			
	Community clinic or health center	8 (38)	1 (25)
	Specialty clinic	13 (61)	3 (75)

**Table 4:** Interviewed participants per case.

Interviewees		Clinic manager, n	Health care providers, n	Nonmedical staff, n	Patients, n	Caregiver, n
**Clinic 1**						
	MyChart user	1 (female)	3 (2 female and 1 male)	0	4 (female)	1 (male)
	MyChart nonuser	0	0	0	0	0
**Clinic 2**						
	MyChart user	1 (female)	5 (female)	0	5 (female)	N/A^a^
	MyChart nonuser	0	0	0	0	0
**Clinic 3**						
	MyChart user	1 (female)	4 (female)	0	4 (3 female and 1 male)	N/A
	MyChart nonuser	0	1 (male)	0	1 (male)	N/A
**Clinic 4**						
	MyChart user	1 (male)	2 (1 female and 1 male)	4 (female)	11 (5 female and 6 male)	N/A
	MyChart nonuser	0	5 (4 female and 1 male)	0	1 (male)	N/A
**Clinic 5**						
	MyChart user	0	0	0	0	N/A
	MyChart nonuser	0	1 (male)	0	0	N/A

^a^N/A: not applicable.

### Themes

We coded each group of interviewees (clinic managers, health care providers, nonmedical staff, and patients) separately. As we were reviewing the transcripts and coding them, it became evident that the concerns raised by nonusers were also raised by users of MyChart. Therefore, information collected from users and nonusers was aggregated. Textbox 1 shows the identified themes.

**Themes per sample group.**

**Patients**

- Theme 1: My health, my responsibility, but I need the information to do that
- Theme 2: Convenience, convenience, convenience
- Theme 3: A few tweaks will do it
- Theme 4: Do not take it away

**Health care providers**

- Theme 1: Of course we need it
- Theme 2: Yes, it did some good things
- Theme 3: Not all was great
- Theme 4: Consider my needs

**Clinic managers**

- Theme 1: Time for some efficiency in communication
- Theme 2: Heard great things about it
- Theme 3: Heard about some issues as well
- Theme 4: Change is needed

**Nonmedical providers**

- Theme 1: Great service for the patient
- Theme 2: Needs some education
- Theme 3: Need to maintain and expand

The themes from each sample group are described in more detail below, with direct quotations used to support the analysis.

#### Patients

A total of 27 patients from 4 clinics were interviewed. Four themes emerged from the interviews: (1) my health, my responsibility, but I need the information to do that; (2) convenience, convenience, convenience; (3) a few tweaks will do it; and (4) do not take it away.

##### Theme 1: My Health, My Responsibility, but I Need the Information To Do That

When initially offered, patients were unsure what to expect from a system that would give them access to their own health care information—information that had been difficult to obtain previously. Furthermore, participants noted that the introduction to MyChart was not as convincing as it might have been, thus they were unsure if there was any benefit for them. Patients decided to enroll and use MyChart based on their interest in being more proactive in their own health:

First, I am convinced that my health is MY responsibility, not the physician or treatment team. They are here to advise and instruct and carry out detailed procedure that I accept. I must be convinced of the efficacy of treatment options. I am not a blind follower of absolute directions from the all-knowing care-teams. I always try to enter into a meaningful dialogue about treatment options and expected results, and I expect the treatment teams to deal with me professionally and personally.

1PAT3

Patients noted that seeing their medical results did not cause them to be anxious or call the doctor’s office more, as had been expected by their health care providers. Patients were genuinely surprised that the system actually maintained records of their health information. Some were surprised to learn things that they were uninformed about previously.

This theme centered on the importance of having access to credible information that “helps me look after me” (3PAT1). Having access to the information allowed patients to make more informed choices. Empowerment and comfort were also considered important:

It has been empowering for me and that also feel that because of it, I am burdening the system a lot less. Fewer appointments, fewer phone calls...and also I feel not just burdening the system but I’m better able to take care of myself.

3PAT1

Patients no longer accepted the modus operandi that *no news, is good news*. They wanted to know whether their results were *good* or *bad*. Having access to their medical records was seen as a necessity, as patients were already trying to collect and maintain it by requesting copies from their health care providers. Patients expressed frustration with the fact that patient portals exist elsewhere and regarded the process of implementation in Alberta to be slow. Overall, patients felt that they needed this information to be responsible for their own health.

##### Theme 2: Convenience, Convenience, Convenience

The term *convenience* was used to describe a missing element in health care and the contribution of MyChart to solving this problem. Regardless of where the patient was, they liked being able to connect with their health care team through MyChart. Just knowing that they could reach out to their health care provider was considered a safety net. Being able to contact their health care provider directly through an email system and receive prompt replies was appreciated. Patients indicated that, from their point of view, they did not abuse this system and they only sent messages when needed. Although patients did not understand all their test results, they saw the benefits of having the information and used it to frame more specific questions for their health care providers and to discuss treatment plans. Most of the time, the provided ranges of results were sufficient for patients to understand their results. Furthermore, some patients had been performing the same tests over the years, thus they were already aware of how to interpret their test results.:

I may not understand everything, in fact I understand very little but this information enables me to ask a smarter question.

4PAT2

Patients wanted the same convenience in interactions with their health care provider as for other services such as banking and shopping. The ability to make and cancel appointments web-based was described as extremely convenient and valuable. One patient explained that “you should not have human resources doing the work that can be done with clicks” (4PAT2). Another patient described the ability to make appointments web-based as *freedom.* In addition, having the ability to fill out questionnaires before going to the appointment was seen as beneficial, as patients had the ability to take the time and carefully think about the answers. Caregivers used the information in MyChart to monitor the health of their family members.

Patients correlated the convenience of MyChart with cost saving, as patients were able to see their results without making unnecessary follow-up appointments, did not have to travel for a short appointment, did not have to take time off from work, and did not have to pay for parking and/or pay to get their results printed. They saw this not only as cost savings to them but also as cost savings to the health care system.

Although proxy access was one of the most challenging elements to set up within MyChart, patients noted that it was one of the most beneficial connections, especially when caring for a family member:

Now my father who is 90 is a different story and one of the very interesting and most important features of this is the availability we have to see dad's test results. So, I can access because you know my dad and I work together, and this is maybe kind of not how they thought this was going to work, but as a primary caregiver with my dad, I’ve got his user ID and password, which I set up for him anyway.

4PAT3

Patients who had provided proxy access to their accounts were cognizant of the possible issues, but they found that the benefits outweighed the risks. Patients in our study did not seem to be greatly concerned about privacy and security. They felt that this system was as secure as any other system that had their personal information:

Well there's a concern every time I pull out my debit card or my visa. There is a concern every time I go on Instagram. There’s a concern with every single thing we do and I could choose to be paralyzed by that and not have a credit card, and not go online, not do anything. But I choose to do all those things to enjoy the benefits, I guess. It's I suppose convenience and you use reasonable care, I think. And you kind of recognize that things can be hacked into and data can be lost.

4PAT4

##### Theme 3: A Few Tweaks Will Do It

Although patients found MyChart very beneficial, they also described some of the barriers that they experienced with the system. Some patients expressed concerns about their ability to understand the results, as they did not find the descriptions in MyChart valuable at all times. Diagnostic reports were not accompanied with explanations; thus it was difficult for patients to interpret the findings. Some patients did not appreciate the fact that health care providers did not see them as sufficiently competent to understand the information:

But you know the medical professionals are God and everybody else bows before them and does whatever they say. You know they're the only ones that can interpret this stuff properly.

4PAT4

Patients expressed dissatisfaction about the inability to see the entire medical record, including the doctor’s notes.

No education or training on how to use MyChart was provided. As they did not know what their use of MyChart would entail, some patients decided not to sign up, noting that it might take “too much time to learn the things” (4PAT11 [NU]). Patients were frustrated about the lack of access to most MyChart functions. Patients in the clinics without the option to book web-based appointments defined this gap as a barrier:

I can book my flights, I can book my hotels, I can do it all on an app on my smartphone. I can book my lab, why can’t I book my doctor's appointment.

4PAT4

Patients also did not understand why all their family members could not be on the system.

The sign-up and the sign-in process were barriers to some patients. One person said, “I mean I understand that they need a certain security level, I totally understand that, but it was so delicate and so picky that it often did’nt work” (4PAT1). Although MyChart was seen as a beneficial tool that enhanced the patient-provider relationship, some expressed concerns about health care being computerized and what that would mean for patients.

##### Theme 4: Do Not Take It Away

Many of the interviewed patients expressed concerns about the possibility that MyChart might not be continued after the pilot and said it would be like going “back to the Stone Ages” (4PAT4). They said they were not being informed on regular bases about the future of MyChart. Furthermore, patients explained that if MyChart was removed, they would have to go back to playing phone tag with their providers:

In this world where we have access to so much information and can make choices and view and control things online. My expectation is that I should be able to do that with my healthcare. And my experience is it's probably giving me better healthcare more efficiently than the old way.

4PAT4

Participants wanted to inform decision makers regarding the value of the patient portal and their wish for it to be maintained, and they had some suggestions for improvements (see Multimedia Appendix 4 for additional quotes).

#### Health Care Providers

A total of 21 health care providers from 5 clinics were interviewed. Four themes emerged from the interviews: (1) of course we need it; (2) yes, it did some good things; (3) not all was great; and (4) consider my needs.

##### Theme 1: Of Course We Need It

We commenced the interviews with health care providers by soliciting opinions about their perceptions of MyChart and their experience of its implementation. Many expressed positive views as they saw it as a good vehicle for delivering information to patients:

I think it's the patient's information. And unless there’s a really clear medical reason, like maybe they have severe anxiety, mental health issues, or something else that maybe they shouldn’t have it. I don’t see any reason why they shouldn’t have that information based on pretty much real time unless there are a certain subset of things that possibly might qualify for that maybe it's a cancer diagnosis.

4HCP1

One described MyChart as “a piece of our healthcare system that's been missing” (1HCP2). Furthermore, many providers agreed that patients had the right to their information and said portals were a great tool for that purpose. Providers thought MyChart had a positive impact on both themselves and their patients, “I can say fairly from my patients that experience is always been positive and been positive to me” (1HCP2).

##### Theme 2: Yes, It Did Some Good Things

Health care providers described several benefits from the MyChart for patients, “by learning about their health even and that sort of thing that they can click on into a health portal and learn about their condition or what comes up, or surgeries or whatever” (1HCP1). Providing patients with information improves patient visits. One provider said, “when you are armed with the results and armed with some of those values, it will make the patient interviews and patient visits a bit more meaningful” (4HCP2). MyChart was seen as a secure system where everything was recorded, as even a nonuser provider stated, “everything's documented so you don't have to worry” (4HCP4 [NU]).

Providers stated that MyChart was desirable as it reduced phone calls and provided an alternative means of communication. One of its novel functions was 2-way secure communication for nonemergency issues. This function required many modifications and decisions because it had not been tested before the pilot, and participating clinicians had expressed skepticism. It was also one of the main reasons many health care providers within the participating clinics decided not to participate in the pilot. Providers assumed that they would be inundated with a high number of messages, and there was no payment plan in place to remunerate them for the time required to answer these messages. As patients on MyChart started calling the clinic less frequently to get results, health care providers recognized the improvement in workflow, “instead of them calling us, they’re just sending us a message through MyChart, which is easier for us on our end” (2HCP4).

Although only 1 clinic during the pilot decided to implement the appointments function for their patients, it proved to be extremely valuable as patients could “do it at their convenience to save some calling back and forth” (4HCP1). Providing proxy access was seen as a much-needed innovation in delivering health care. It was a new function that was never offered in Alberta before the MyChart pilot. Providers were positive about the approach. The most common request for proxy was for a spouse or adults caring for their elderly parents. One provider said, “the wife is the one that like keeps track of all the health information or like the different results and medications and whatnot so they’re usually the ones that email about their husband's symptoms or questions or whatnot” (2HCP1).

MyChart did not impact workflow negatively, as was originally expected. The major benefits in terms of workflow was that it improved communication between patients and providers and that it provided a means of accessing information quickly. The work itself did not change, but there was a change in the way the work was performed. The system allowed other team members to be informed about communication and decisions.

##### Theme 3: Not All Was Great

Although there were many benefits of having MyChart available to patients, providers expressed several concerns. Not all patients were deemed fit to have access to their health care information, as “too much information sometimes is not a great thing” (1HCP1). Another provider stated that “a lot of patients wouldn’t really want to know that; they’ll just want to know if they’re living or dying” (5HCP1 [NU]). Providers made decisions about which patients would be offered MyChart and stated that sometimes they did not offer it because there was no time to do so:

I offer it to all although I can tell you I'm not consistent. So, I mean to offer it all but there's plenty of times when I’m rushing around in a clinic, and/or if a person has a lot of other concerns it doesn’t pop into your mind and so you’re more focused on other things during your clinic visit.

2HCP3

Health care providers did not want the additional work of educating and helping patients or providing technical support. One provider stated, “more physicians are annoyed with that; they’ll just stop offering that service” (1HCP3). This reluctance was based on previous experiences that technologies tend to be implemented without proper technical support or education. Providers felt that they had to *wing it* in using the system. MyChart was perceived as potentially taking time away from medical consultations if providers had to do promotion and education. As another provider stated, “I do not have time in that time to introduce a concept that has to be done by somebody else” (3HCP5). Some providers described that they just forgot about MyChart and did not bother with enrolling patients:

It's kind of forgotten sometimes and it could be the nature of the clinic. It’s a busy clinic and maybe it's just forgotten as a tool. There is one physician that does use it more so than others.

3HCP3 NU

There were no clear guidelines and expectations, thus there was hesitancy about what was the right thing to do. Health care providers expressed apprehension because they were unclear what MyChart meant in terms of changes in charting practices.

##### Theme 4: Consider My Needs

When asked about the upcoming provincial-wide implementation, health care providers stated that if MyChart were continued, then it needed to reflect their needs and be established in a manner that would work within their busy schedules. It was stated that the system would need to be developed around some kind of incentives. No incentives would mean that “people won’t use it” (1HCP3) and patients would not receive the opportunity to be introduced to the portal. Policies and guidelines should be implemented about charting, proxy access, and 2-way communication. MyChart could be incorporated as long as it did not contribute to additional workload (see Multimedia Appendix 4 for additional quotes).

#### Clinic Managers

A total of 4 clinic managers were interviewed, and 4 themes emerged from the interviews: (1) time for some efficiency, (2) heard great things about it, (3) heard about some issues as well, and (4) culture change is needed.

##### Theme 1: Time for Some Efficiency in Communication

When managers were asked why they decided to be part of the pilot, they stated that it was to eliminate paper and phone calls, try an innovation, and provide new ways for patients to manage complex chronic conditions. They had positive views about portals and how portals could impact practice in a positive way. This was an important finding, as clinic managers were involved in decisions about whether to have their clinic involved in the pilot:

I think that you know this is going to be the future. The question is how we get there.

4CM1

They viewed MyChart as a way to connect team members and assist patients and providers. They thought that the system had the capacity to improve the communication processes currently broken in the health care system, as patients continue to complain that they are unable to reach their providers when needed.

##### Theme 2: Heard Great Things About It

Clinic managers stated that the impact of patients seeing their medical records was seen as a facilitator to better care. Patients already search for things on the web, and MyChart provided access to correct and legitimate information:

Our patients are different patients than what they were 20 or 30 years ago because they have Internet access already. They already research all.

2CM21

It is important to note that clinic managers agreed that not all patients would use MyChart and that the system needed to accommodate this possibility. The MyChart 2-way communication function capacity was seen as a means of establishing new ways of communication and reducing reliance on phone calls.

##### Theme 3: Heard About Some Issues As Well

When the pilot started, only physicians were allowed to sign up patients to MyChart. This led to low enrollment numbers because physicians did not have enough time to explain MyChart to each patient. Furthermore, clinic managers described that many physicians did not participate in the pilot even though the clinic was part of the pilot. This was because of a lack of buy-in as physicians did not see MyChart as something beneficial. One clinic manager identified the issue of control as a drawback to the use of MyChart:

Physicians traditionally are so accustomed to kind of controlling everything that happens with that patient. And when the patient starts seeing the results of what they’re trying to achieve. They're worried about it derailing the treatment progress and the relationship between the provider and the patient, when in fact I think the actual opposite occurs.

2CM21

Clinic managers acknowledged that they heard from the health care providers about patients having issues with MyChart. The sign-up process was challenging and created grievances. Technical support was lacking, and health care providers had to figure out ways to help patients with the sign-up process. One of the barriers was that physicians did not take full responsibility for the 2-way communication.

##### Theme 4: Change Is Needed

Clinic managers stated that change is needed in the current provider-patient relationship. They agreed that all clinics should be able to provide it, so all patients would have the same opportunity to access their health care information:

It needs to be a normal part of the life or not, it needs to be a normal part of, more the norm than the exception.

1CM1

Furthermore, clinic managers stated that physicians need to learn how to work with innovations such as MyChart and that these changes might not be easy in the beginning but are needed (see Multimedia Appendix 4 for additional quotes).

#### Nonmedical Staff

Only 1 clinic within the pilot opened the web-based appointment booking and canceling appointments function. For the scheduling function to be accessible, participating health care providers had to enter their availability in the system, which would allow patients to select from the available slots. Once a patient selects a slot, the clinic receives a message. If the booking was done incorrectly or the slot was changed, the office staff would call the patient and modify the booking. From these interviews, we identified 3 themes: (1) great service for the patients, (2) needs some education, and (3) need to maintain and expand.

##### Theme 1: Great Service for the Patient

The nonmedical staff described that participants “loved booking their own appointments especially because they know (if) something happens in the middle of the night, they can go on and book for the next day if there's openings” (4NON2). If there were any issues with the appointment, then the staff would call the patient and get it all sorted out; therefore, MyChart lightened the workload.

##### Theme 2: Needs Some Education

As not all health care providers participated in the pilot, some patients in a given clinic were able to make web-based appointments, whereas others could not do so. In addition, a challenge was with patients who would book many appointments, as “they can book a whole bunch of them on MyChart in one day” (04MANON00213DEC2018).

##### Theme 3: Need to Maintain and Expand

Staff indicated that they would like more information about MyChart so that they could assist patients better and said that they needed “more awareness and if they ask questions I can help as I can't help them much now” (4NON3; see Multimedia Appendix 4 for additional quotes).

### Summary of the Results

We report the uptake and adoption of an initial phase of a patient portal introduction among 5 health care clinics that were, at the outset of the introduction, judged to be clinics where high uptake would be found. In contrast, 2 clinics showed high uptake, 1 clinic showed moderate uptake, 1 clinic showed low uptake, and the last clinic was categorized as a clinic without use. We identified several factors related to the uptake of the patient portal, including satisfaction and/or dissatisfaction with the current communication tools, performance expectancy, facilitating conditions, behavioral intentions, and use behavior. All these factors had an impact on the level of uptake in each setting and created different opportunities for the end users. Table 5 summarizes our findings about each clinic by considering our findings.

**Table 5:** Impact of the MyChart pilot on each case study.

Case study	Level of usage	Satisfaction or dissatisfaction with the current communication tools	Performance expectancy	Facilitating conditions	Behavioral intention	Use behavior
Clinic 4	High user; 965 enrolled patients	Clinic considered as an innovation hub clinic; first clinic to go live within the PoC^a^; 2 family doctors and a registered nurse participated; 3 family doctors did not participate	MyChart described as the *most rewarding* initiative; offered the web-based booking and canceling appointment function. Only clinic to do so	The registered nurse acted as the MyChart support person	High enrollment as they offered it to almost all patients	The clinic maintained high enrollment and incorporated the frontline staff (office assistants) to be part of the process
Clinic 2	High user; 875 enrolled patients	It was described that the clinic had the *old way* and the *new way* processes	The participating providers used the technology to share medical results with their patients and decrease the number of phone calls to the clinic	Two specialists maintained the *old way* who did not participate in the PoC because of the assumption that it will increase their workload and patients will contact them all the time. Two specialists implemented the *new way*	This process was maintained for a while, until the nurses from the nonparticipating specialists decide to start enrolling patients on MyChart	After the nurses took on the initiative to sign up patients to MyChart, the enrollment increased
Clinic 1	Medium user; 357 enrolled patients	MyChart was brought in because of the potential for uploading forms and questionnaires that patients tend to do	The technology did not produce the identified needs as the forms on MyChart were found to be inappropriate by the clinic	Nurses took on the active role in enrolling patients	Only offered to certain patients (medium enrollment)	The implementation process was observed by 2 other specialty clinics that enrolled in the PoC and achieved a high enrollment (1047 and 667 patients)
Clinic 3	Low user; 172 enrolled patients	The participating specialist is the clinic manager that brought MyChart to the clinic with the intent to decrease the number of phone calls or unnecessary follow-up appointments	The portal was deemed needed because of the 2-way communication function. However, patient messages sent through MyChart were often unanswered because of staff changes	The nurses did not actively participate in the enrollment process; other staff did not see the benefit and were unwilling to participate because of the 2-way communications function	Low enrollment, although patients expressed interest (interviewed 1 patient from this clinic who did not have access but wanted it)	MyChart was described as an *afterthought*
Clinic 5	Nonuser; 0 enrolled patients	Although offered to participate, the staff at this academic setting family clinic decided not to	The technology was deemed difficult to incorporate in the clinic’s flow	The clinic has patients with MyChart because of access provided at other clinics, but no provider from this clinic wanted to use or assist patients with the portal	The portal was not seen as something necessary in the delivery of health care services	No perceived value and thus no enrolment or usage

^a^PoC: proof of concept.

## Discussion

### Principal Findings

In our study, dissatisfaction with current health care communication tools appeared to be the primary factor that drove patients’ and providers’ decisions to consider the use of MyChart. Once they had made this decision, uptake was dependent on performance expectancy, which included understanding how to use the technology and valuing the additional functionality that it offered. Furthermore, although the themes emerged from the interview data, they aligned with the *theory of technology acceptance* by Davis [14] and included *perceived usefulness*, *perceived ease of use*, *barriers to ease of use*, and *future usage* [14]. For example, theme 1 from the patient interviews was *my health, my responsibility, but I need the information to do that*, which correlated with *perceived usefulness* [14], as shown in Textbox 2.

**Themes per sample group correlating with the theory of technology acceptance.**

**Patients**

- Theme 1: My health, my responsibility, but I need the information to do that (perceived usefulness)
- Theme 2: Convenience, convenience, convenience (perceived ease of use)
- Theme 3: A few tweaks will do it (barriers to ease of use)
- Theme 4: Do not take it away (future usage)

**Health care providers**

- Theme 1: Of course we need it (perceived usefulness)
- Theme 2: Yes, it did some good things (perceived ease of use)
- Theme 3: Not all was great (barriers to ease of use)
- Theme 4: Consider my needs (future usage)

**Clinic managers**

- Theme 1: Time for some efficiency in communication (perceived usefulness)
- Theme 2: Heard great things about it (perceived ease of use)
- Theme 3: Heard about some issues as well (barriers to ease of use)
- Theme 4: Change is needed (future usage)

**Nonmedical providers**

- Theme 1: Great service for the patient (perceived usefulness)
- Theme 2: Needs some education (barriers to ease of use)
- Theme 3: Need to maintain and expand (future usage)

The conditions that facilitated understanding of the technology varied across clinics. Some clinics formally designated assistance with MyChart use to staff in their clinic. All clinics had high or medium uptake of MyChart. In other clinics, staff decided not to offer MyChart to some patients, which had an impact on the uptake of MyChart. Reasons are not entirely clear and require further investigation. Some patients who understood how to use the technology and valued it became *users*, whereas others did not. The main reason for discontinuing use appeared to be the lack of an immediate health care concern [25].

**Figure 2:**
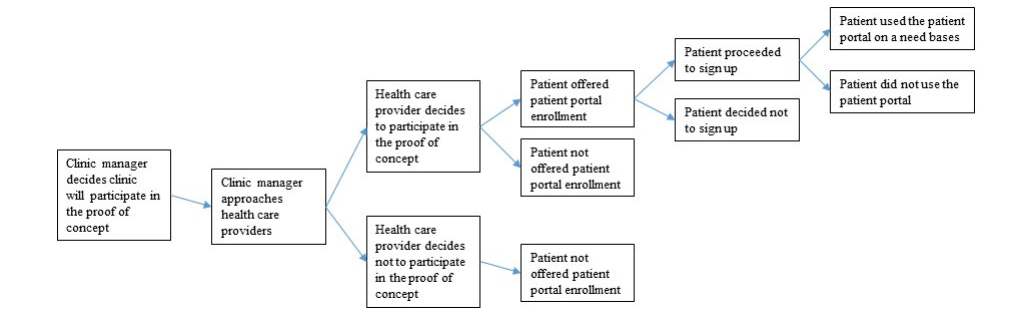
Clinic managers as gatekeepers to uptake of MyChart.

Clinic managers were foundational to the uptake of MyChart (Figure 2), a finding that has not been discussed in the literature to date. They made decisions about whether to present the technology to providers. Our data suggest that this decision was based on an informal assessment of satisfaction with the current communication tools. Clinic managers who declined participation in the pilot indicated that their clinic patients and providers would not be interested in trying the technology. Figure 2 summarizes the impact of the clinic managers on the uptake of MyChart.

The actions of the clinic managers could be observed through Roger’s theory of diffusion, as they served as early adopters by assessing the advantages or disadvantages of the innovation. They determined the *perceived efficiencies*, its *compatibility* with the existing system, the *complexity*, and, ultimately, the *benefits* and unintended *consequences* of the innovation [13].

Usage and enrollment depended on how patients were informed about the portal. Patients in this study reported variable understanding of the portal following the introduction by clinic staff. Similar to the study by Kim et al [25], a quick introduction did not appear to be valuable to patients. In this study, participating health care providers reported not having sufficient time to explain the portal to patients. In clinics with higher uptake, a short introduction became incorporated into the clinic visit workflow, which allowed for more sufficient means in enticing patients to sign up.

Knowledge about the technology was another important component that led to increased or decreased usage. Ryan et al [26] found that patients and health care providers had positive views about patient portals but that older patients had less knowledge about patient portals. In this study, older participants reported that they took the time to learn and understand the system once they decided to enroll. We found that patients had to learn the system by *trial and error*. When patients were unable to navigate the sign up and authentication, the use of MyChart became impossible. When patients accessed medical information in MyChart, they reported becoming more active in the management of their health, which allowed them to see their health trends and identify opportunities for self-care. Patients came prepared with specific health questions because they could spend less time going over less valuable health information (eg, lab results) and making better use of scarce time with their care provider. This finding was similar to the results reported by Dendere et al [27].

Some research teams have reported a relationship between concerns regarding privacy and technology uptake [28-36]. In our study, participants did not report perceiving significant risks to having the confidentiality of their health information violated. Participants reported a sense that the health system would maintain appropriate controls and procedures to protect the security of their health information.

Although not all health care providers saw the *value* of patients having access to the same information, participants *championing* use of MyChart experienced more efficient ways to connect and communicate with their patients. This finding is in contrast to the findings reported by others who have studied patient portals [27,37-40] and reported that portals increased workload. For example, Koivunen et al [41] described the negative views of nurses about patient portals. Our findings are consistent with the work of other research teams who reported that nurses saw patient portals as an opportunity to improve communication with patients [42-44].

Furthermore, theories of how patient portals support health care system transformation may not draw upon crucial empirical data. The current findings emphasize that without a shared understanding of the purpose of patient portals and some dissatisfaction with existing communication methods, uptake lags. We posit that these aspects will be crucial as patient portals introduced in real-life care settings often accompany other technologies for clinicians and managers to navigate [44].

We agree with Wiljer et al [45], who described a need for a culture shift, where access to medical records is a fundamental right of every patient. A shift of this magnitude could only be accomplished with broad support from policy makers, health care providers, health administrators, and patients. Without adequate support for patients as complete partners in the management of their health, patients would be left to take actions based on incomplete and possibly inaccurate information from untrustworthy sources. Clinically useful uptake of patient portals is an essential enabler for patients as partners in managing their health and transforming the wider health system.

### Limitations

There were several limitations to this study. Patient interviews were only conducted with patients who volunteered to participate and were attending an appointment with a health care provider who was also participating in the study. The information provided by participants may not have been recalled accurately by them. In addition, although all efforts were made, we were unable to identify and recruit participants who did not continue to use MyChart as we had no means to obtain information about discontinued usage. Finally, the findings should be generalized with caution to other health care systems and to settings that use other types of patient portals.

### Conclusions

This work exposes a number of factors that have an impact on the uptake of MyChart. We found that dissatisfaction with nonportal-based communications was a foundational element that likely needs to be addressed before more advanced goals can be reached. We also found that clinical managers were key gatekeepers in the uptake process. Once a clinic manager decided that the clinic should use the portal, the designation of a staff member to help patients become more familiar with the portal appeared to increase uptake.

## Appendix

Multimedia Appendix 1 Recruitment card.

Multimedia Appendix 2 Comparative case interview guide questions.

Multimedia Appendix 3 Interview codes for all interviewed participants in each case setting.

Multimedia Appendix 4 Additional quotes supporting thematic analysis.

## Abbreviations

AHS: Alberta Health Services
CIHR: Canadian Institutes of Health Research
