# Supplementary material for: Patient Portal Implementation and Uptake: Qualitative Comparative Case Study
Source: J Med Internet Res. 2020 Jul 27;22(7):e18973. doi: 10.2196/18973 (PMC7427986; doi:10.2196/18973)
Supplement: Multimedia Appendix 2 [file jmir_v22i7e18973_app2.docx]

## Appendix 2: Comparative Case Interview Guide Questions

Thank you for agreeing to meet with me today. As noted in the consent form, your participation is entirely voluntary, and the interview will take approximately 30 minutes.

### Patient Interview Guide Questions

As mentioned in the letter attached to the consent form for this study, I am interested in learning more about why you used or did not use the MyChart® portal, and what were your experiences.

Where you offered information about the patient portal MyChart®?

If YES:

Did you sign up as per information material?

If YES:

Can you please tell me about your introduction to the portal?

How and why did you use it?

What did you like?

What you didn’t like?

What would you change?

What would you recommend that it should be done in regards to the portal?

Is there anything else you would like to add and you did not get a chance?

If NO:

Why you didn’t sign up?

What would have made a difference in you signing up for the portal?

What would you recommend that it should be done in regards to the portal?

Is there anything else you would like to add and you did not get a chance?

If NO:

What is your opinion about having access to your own electronic medical record?

What would that do for you?

What you want to be available in healthcare that will help you as a patient?

### Healthcare Provider Interview Guide Questions

As mentioned in the letter attached to the consent form for this study, I am interested in learning more about your perceptions and experiences regarding the benefits or limitations of MyChart® in the patient/healthcare provider relationship, strategies for assessing the benefits and risk of implementing MyChart®, information input and sharing, data security and confidence in the information, and reporting by patients about lifestyle choices and behaviours.

1. Tell me about your perspective on patient portals?
2. What a ‘good’ patient portal should do?
3. How has the MyChart® Proof of Concept impacted your practice? (Positive and negative consequences)
4. What about sharing sensitive information with your patients?
5. What patient’s feedback did you receive?
6. What do you wish was done differently?
7. What improvements would you recommend to be incorporated into MyChart® prior to the provincial implementation?
8. Is there anything else you would like to add and you did not get a chance?

### Administrator/Manager Interview Guide Questions

As mentioned in the letter attached to the consent form for this study, I am interested in learning more about the plans and barriers to adoption and problem-solving approaches related to MyChart®.

1. Tell me about your perspective on patient portals?
2. What a ‘good’ patient portal should do?
3. Why did you decide to participate in the MyChart® Proof Concept?
4. How has the MyChart® Proof of Concept impacted your practice?
5. What do you wish was done differently?
6. What patient’s feedback did you receive?
7. What improvements would you recommend to be incorporated into MyChart® prior to the provincial implementation?
8. Is there anything else you would like to add and you did not get a chance?

### Non-medical Providers Interview Guide Questions

As mentioned in the letter attached to the consent form for this study, I am interested in learning more about how your clinic implemented the online appointment booking and canceling option.

1. Tell me how was the booking function introduced at your clinic?
2. How patients used the functions?
3. What were some of the facilitators from having the function available to patients?
4. What were some of the barriers from having the function available to patients?
5. What would you recommend for improvement?

## Appendix 3: Interview codes for all interviewed participants per case setting

| **Case Study** | **Type of Participant** | **Interview Code** |
| --- | --- | --- |
| Case 1 (medium user) | Clinic Manager | 1CM1 |
|  | Healthcare Providers | 1HCP1 |
|  |  | 1HCP2 |
|  |  | 1HCP3 |
|  | Patients | 1PAT1 |
|  |  | 1PAT2 |
|  |  | 1PAT3 |
|  |  | 1PAT4 |
|  |  | 1PAT5 |
|  |  | 1PAT6 |
|  | Caregiver | 1CGP1 |
| Case 2 (high user) | Clinic Manager | 2CM21 |
|  | Healthcare Providers | 2HCP1 |
|  |  | 2HCP2 |
|  |  | 2HCP3 |
|  |  | 2HCP4 |
|  |  | 2HCP5 |
|  | Patients | 2PAT1 |
|  |  | 2PAT2 |
|  |  | 2PAT3 |
|  |  | 2PAT4 |
|  |  | 2PAT5 |
| Case 3 (low user) | Clinic Manager | 3CM1 |
|  | Healthcare Providers | 3HCP1 |
|  |  | 3HCP2 |
|  |  | 3HCP3(NU)* |
|  |  | 3HCP4 |
|  |  | 3HCP5 |
|  | Patients | 3PAT1 |
|  |  | 3PAT2 |
|  |  | 3PAT3 |
|  |  | 3PAT4(NU) |
|  |  | 3PAT5 |
| Case 4 (high user) | Clinic Manager | 4CM1 |
|  | Healthcare Providers | 4HCP1 |
|  |  | 4HCP2 |
|  |  | 4HCP3(NU) |
|  |  | 4HCP4(NU) |
|  |  | 4HCP5(NU) |
|  |  | 4HCP6(NU) |
|  |  | 4HCP7(NU) |
|  | Patients | 4PAT1 |
|  |  | 4PAT2 |
|  |  | 4PAT3 |
|  |  | 4PAT4 |
|  |  | 4PAT5 |
|  |  | 4PAT6 |
|  |  | 4PAT7 |
|  |  | 4PAT8 |
|  |  | 4PAT9 |
|  |  | 4PAT10 |
|  |  | 4PAT11(NU) |
|  |  | 4PAT12 |
|  | Non-medical Providers | 4NON1 |
|  |  | 4NON2 |
|  |  | 4NON3 |
|  |  | 4NON4 |
| Case 5 (non-user) | Healthcare Provider | 5HCP1(NU) |
| *(NU) means non-user | |  |

## Appendix 4: Interview participants demographic information

| **Patient demographics (n=27)** | | |
| --- | --- | --- |
| Characteristics | n | (%) |
| **Gender** |  |  |
| Female | 17 | (62.96%) |
| Male | 10 | (37.03%) |
| **Age** |  |  |
| 18 to 25 | 1 | (3.70%) |
| 26 to 35 | 1 | (3.70%) |
| 36 to 45 | 0 | (0.00%) |
| 46 to 60 | 16 | (59.25%) |
| 61 and over | 9 | (33.33%) |
| **Marital Status** |  |  |
| Never legally married | 2 | (7.40%) |
| Legally married (and not separated) | 18 | (66.66%) |
| Separated, but still legally married | 0 | (0.00%) |
| Divorced | 2 | (7.40%) |
| Common law | 4 | (14.81%) |
| Widowed | 1 | (3.70%) |
| **Education Level** |  |  |
| Less than high school degree | 0 | (0.00%) |
| High school degree or equivalent | 5 | (18.51%) |
| Some post-secondary education but no degree | 7 | (25.92%) |
| Registered Apprenticeship or other trades certificate or diploma | 5 | (18.51%) |
| Associate degree | 1 | (3.70%) |
| Bachelor degree | 7 | (25.92%) |
| Graduate degree | 0 | (0.00%) |
| Post-graduate degree | 2 | (7.40%) |
| **Employment Status** |  |  |
| Employed, working 40 or more hours per week | 9 | (33.33%) |
| Employed, working 1-39 hours per week | 7 | (25.92%) |
| Not employed, looking for work | 0 | (0.00%) |
| Not employed, NOT looking for work | 2 | (7.40%) |
| Retired | 4 | (14.81%) |
| Unable to work | 3 | (11.11%) |
| Self-Employed | 2 | (7.40%) |
| **Family Income** |  |  |
| Less than $20,000 | 0 | (0.00%) |
| $20,000 to $34,999 | 1 | (3.70%) |
| $35,000 to $49,999 | 2 | (7.40%) |
| $50,000 to $74,999 | 1 | (3.70%) |
| $75,000 to $99,999 | 4 | (14.81%) |
| $100,000 to $149,999 | 8 | (29.62%) |
| $150,000 or More | 11 | (40.74%) |
| **Chronic Condition** |  |  |
| Yes | 23 | (85.18%) |
| No | 4 | (14.81%) |
| **MyChart® Users** |  |  |
| Yes | 25 | (92.59%) |
| No | 2 | (7.40%) |
| And/or Proxy | 5 | (18.51%) |

| **Healthcare Providers (n=21) and Clinic Managers (n=4) demographics** | | | | |
| --- | --- | --- | --- | --- |
|  | **Healthcare providers demographics (n=21)** | | **Clinic Manager demographics (n=4)** | |
| Characteristics | n | (%) | n | (%) |
| **Gender** |  |  |  |  |
| Female | 16 | (76.19%) | 3 | (75.00%) |
| Male | 5 | (23.80%) | 1 | (25.00%) |
| **Age** |  |  |  |  |
| Over 18 | 0 | (0.00%) | 0 | (0.00%) |
| 18 to 29 | 0 | (0.00%) | 0 | (0.00%) |
| 30 to 39 | 2 | (9.52%) | 0 | (0.00%) |
| 40 to 49 | 8 | (38.09%) | 2 | (50.00%) |
| 50 to 59 | 8 | (38.09%) | 1 | (25.00%) |
| 60 to 64 | 2 | (9.52%) | 1 | (25.00%) |
| 65 and over | 1 | (4.76%) | 0 | (0.00%) |
| **Type of** |  |  |  |  |
| Family physician | 2 | (9.52%) | 1 | (25.00%) |
| Family physician with a focused practice | 0 | (0.00%) | 0 | (0.00%) |
| Specialty physician | 5 | (23.80%) | 2 | (50.00%) |
| Registered nurse | 8 | (38.09%) | 1 | (25.00%) |
| Registered practical nurse | 0 | (0.00%) | 0 | (0.00%) |
| Licensed practical nurse | 0 | (0.00%) | 0 | (0.00%) |
| Registered psychiatric nurse | 0 | (0.00%) | 0 | (0.00%) |
| Mental health nurse | 0 | (0.00%) | 0 | (0.00%) |
| Nurse practitioner | 1 | (4.76%) | 0 | (0.00%) |
| Nurse educator | 0 | (0.00%) | 0 | (0.00%) |
| Medical office assistant | 4 | (19.04%) | 0 | (0.00%) |
| Resident | 1 | (4.76%) | 0 | (0.00%) |
| **Working at** |  |  |  |  |
| Academic based care | 14 | (66.66%) | 3 | (75.00%) |
| Community based care | 7 | (33.33%) | 0 | (0.00%) |
| Both | 0 | (0.00%) | 0 | (0.00%) |
| **In which type of setting do you work:** |  |  |  |  |
| Primary care office | 0 | (0.00%) | 0 | (0.00%) |
| Community clinic/health centre | 8 | (38.09%) | 1 | (25.00%) |
| Public health clinic | 0 | (0.00%) | 0 | (0.00%) |
| Specialty clinic | 13 | (61.90%) | 3 | (75.00%) |
| Hospital | 0 | (0.00%) | 0 | (0.00%) |

| **Non-medical providers demographics (n=4)** | | |
| --- | --- | --- |
| Characteristics | n | (%) |
| **Gender** |  |  |
| Female | 4 | (100.00%) |
| Male | 0 | (0.00%) |
| **Age** |  |  |
| Over 18 | 0 | (0.00%) |
| 18 to 29 | 1 | (25.00%) |
| 30 to 39 | 1 | (25.00%) |
| 40 to 49 | 0 | (0.00%) |
| 50 to 59 | 0 | (0.00%) |
| 60 to 64 | 0 | (0.00%) |
| 65 and over | 2 | (50.00%) |
| **Work setting** |  |  |
| Academic based care |  |  |
| Community based care | 4 | (100.00%) |
| Both | 0 | (0.00%) |
