# Supplementary material for: Patient Portal Implementation and Uptake: Qualitative Comparative Case Study
Source: J Med Internet Res. 2020 Jul 27;22(7):e18973. doi: 10.2196/18973 (PMC7427986; doi:10.2196/18973)
Supplement: Multimedia Appendix 4 [file jmir_v22i7e18973_app4.docx]

## Appendix 4: Interview participants demographic information

| **Patient demographics (n=27)** | | |
| --- | --- | --- |
| Characteristics | n | (%) |
| **Gender** |  |  |
| Female | 17 | (62.96%) |
| Male | 10 | (37.03%) |
| **Age** |  |  |
| 18 to 25 | 1 | (3.70%) |
| 26 to 35 | 1 | (3.70%) |
| 36 to 45 | 0 | (0.00%) |
| 46 to 60 | 16 | (59.25%) |
| 61 and over | 9 | (33.33%) |
| **Marital Status** |  |  |
| Never legally married | 2 | (7.40%) |
| Legally married (and not separated) | 18 | (66.66%) |
| Separated, but still legally married | 0 | (0.00%) |
| Divorced | 2 | (7.40%) |
| Common law | 4 | (14.81%) |
| Widowed | 1 | (3.70%) |
| **Education Level** |  |  |
| Less than high school degree | 0 | (0.00%) |
| High school degree or equivalent | 5 | (18.51%) |
| Some post-secondary education but no degree | 7 | (25.92%) |
| Registered Apprenticeship or other trades certificate or diploma | 5 | (18.51%) |
| Associate degree | 1 | (3.70%) |
| Bachelor degree | 7 | (25.92%) |
| Graduate degree | 0 | (0.00%) |
| Post-graduate degree | 2 | (7.40%) |
| **Employment Status** |  |  |
| Employed, working 40 or more hours per week | 9 | (33.33%) |
| Employed, working 1-39 hours per week | 7 | (25.92%) |
| Not employed, looking for work | 0 | (0.00%) |
| Not employed, NOT looking for work | 2 | (7.40%) |
| Retired | 4 | (14.81%) |
| Unable to work | 3 | (11.11%) |
| Self-Employed | 2 | (7.40%) |
| **Family Income** |  |  |
| Less than $20,000 | 0 | (0.00%) |
| $20,000 to $34,999 | 1 | (3.70%) |
| $35,000 to $49,999 | 2 | (7.40%) |
| $50,000 to $74,999 | 1 | (3.70%) |
| $75,000 to $99,999 | 4 | (14.81%) |
| $100,000 to $149,999 | 8 | (29.62%) |
| $150,000 or More | 11 | (40.74%) |
| **Chronic Condition** |  |  |
| Yes | 23 | (85.18%) |
| No | 4 | (14.81%) |
| **MyChart® Users** |  |  |
| Yes | 25 | (92.59%) |
| No | 2 | (7.40%) |
| And/or Proxy | 5 | (18.51%) |

| **Healthcare Providers (n=21) and Clinic Managers (n=4) demographics** | | | | |
| --- | --- | --- | --- | --- |
|  | **Healthcare providers demographics (n=21)** | | **Clinic Manager demographics (n=4)** | |
| Characteristics | n | (%) | n | (%) |
| **Gender** |  |  |  |  |
| Female | 16 | (76.19%) | 3 | (75.00%) |
| Male | 5 | (23.80%) | 1 | (25.00%) |
| **Age** |  |  |  |  |
| Over 18 | 0 | (0.00%) | 0 | (0.00%) |
| 18 to 29 | 0 | (0.00%) | 0 | (0.00%) |
| 30 to 39 | 2 | (9.52%) | 0 | (0.00%) |
| 40 to 49 | 8 | (38.09%) | 2 | (50.00%) |
| 50 to 59 | 8 | (38.09%) | 1 | (25.00%) |
| 60 to 64 | 2 | (9.52%) | 1 | (25.00%) |
| 65 and over | 1 | (4.76%) | 0 | (0.00%) |
| **Type of** |  |  |  |  |
| Family physician | 2 | (9.52%) | 1 | (25.00%) |
| Family physician with a focused practice | 0 | (0.00%) | 0 | (0.00%) |
| Specialty physician | 5 | (23.80%) | 2 | (50.00%) |
| Registered nurse | 8 | (38.09%) | 1 | (25.00%) |
| Registered practical nurse | 0 | (0.00%) | 0 | (0.00%) |
| Licensed practical nurse | 0 | (0.00%) | 0 | (0.00%) |
| Registered psychiatric nurse | 0 | (0.00%) | 0 | (0.00%) |
| Mental health nurse | 0 | (0.00%) | 0 | (0.00%) |
| Nurse practitioner | 1 | (4.76%) | 0 | (0.00%) |
| Nurse educator | 0 | (0.00%) | 0 | (0.00%) |
| Medical office assistant | 4 | (19.04%) | 0 | (0.00%) |
| Resident | 1 | (4.76%) | 0 | (0.00%) |
| **Working at** |  |  |  |  |
| Academic based care | 14 | (66.66%) | 3 | (75.00%) |
| Community based care | 7 | (33.33%) | 0 | (0.00%) |
| Both | 0 | (0.00%) | 0 | (0.00%) |
| **In which type of setting do you work:** |  |  |  |  |
| Primary care office | 0 | (0.00%) | 0 | (0.00%) |
| Community clinic/health centre | 8 | (38.09%) | 1 | (25.00%) |
| Public health clinic | 0 | (0.00%) | 0 | (0.00%) |
| Specialty clinic | 13 | (61.90%) | 3 | (75.00%) |
| Hospital | 0 | (0.00%) | 0 | (0.00%) |

| **Non-medical providers demographics (n=4)** | | |
| --- | --- | --- |
| Characteristics | n | (%) |
| **Gender** |  |  |
| Female | 4 | (100.00%) |
| Male | 0 | (0.00%) |
| **Age** |  |  |
| Over 18 | 0 | (0.00%) |
| 18 to 29 | 1 | (25.00%) |
| 30 to 39 | 1 | (25.00%) |
| 40 to 49 | 0 | (0.00%) |
| 50 to 59 | 0 | (0.00%) |
| 60 to 64 | 0 | (0.00%) |
| 65 and over | 2 | (50.00%) |
| **Work setting** |  |  |
| Academic based care |  |  |
| Community based care | 4 | (100.00%) |
| Both | 0 | (0.00%) |
